# Development of a Complex Intervention for Perinatal Bladder Management Among Pregnant Women Based on the Medical Research Council Framework: A Development Study

**DOI:** 10.3390/healthcare14213773

**Published:** 2026-11-04

**Authors:** Jingjing Gong, Junying Hao, Lili Xing, Yan Liu, Sen Li

**Affiliations:** Department of Obstetrics and Gynecology, Peking University People’s Hospital, Beijing 100044, China; gongjingjing@pkuph.edu.cn (J.G.); ying19890616@163.com (J.H.); 13522330693@126.com (Y.L.)

**Keywords:** perinatal care, bladder management, postpartum urinary retention, complex intervention

## Abstract

**Highlights:**

**What are the main findings?**

- A theory-informed and evidence-based perinatal bladder management intervention was developed.
- The MRC framework guided integration of evidence, TDF findings, and expert consensus.
- The final protocol includes four domains and 29 intervention components. Health education is integrated as a cross-cutting theme.

**What are the implications of the main findings?**

- The intervention provides a structured pathway for preventing and managing PUR.
- It supports early identification, standardized assessment, and continuity of bladder care.
- It offers actionable guidance for multidisciplinary perinatal teams.

**Abstract:**

Background/Objectives: Postpartum urinary retention (PUR) is a common childbirth complication that may lead to bladder dysfunction and adverse maternal outcomes if not promptly identified and managed. Although existing guidelines address aspects of perinatal bladder care, a standardized, context-specific intervention is still lacking. This study aimed to develop a comprehensive perinatal bladder management intervention for pregnant women using the Medical Research Council (MRC) framework for complex interventions. Methods: This intervention development study was conducted in accordance with the development phase of the updated MRC framework. A multidisciplinary group was established, including experts in obstetrics, anesthesia, midwifery, nursing management, and evidence-based practice. The intervention was informed by a review of the physiological mechanisms underlying postpartum voiding dysfunction and a qualitative study guided by the Theoretical Domains Framework (TDF), involving 16 women after vaginal delivery and 18 healthcare professionals. Evidence was identified and appraised from clinical guidelines, evidence summaries, systematic reviews, expert consensus statements, and randomized controlled trials. A two-round Delphi consultation with 18 experts was conducted to refine and validate the intervention framework. Results: Twelve evidence sources were included: four guidelines, three evidence summaries, three expert consensus documents, one systematic review, and one randomized controlled trial. Thirty-four evidence-based recommendations were synthesized across four domains: antenatal bladder management, intrapartum bladder management, postpartum bladder management, and management of urinary retention. Health education was integrated as a cross-cutting theme. After two Delphi rounds, the final intervention comprised four primary domains and 29 components. Both Delphi rounds achieved 100% response rates, with high expert authority coefficients (Cr = 0.956 and 0.972). Conclusions: Using the MRC framework, this study developed a theory-informed and evidence-based perinatal bladder management intervention integrating evidence synthesis, clinical expertise, and stakeholder perspectives. The intervention provides a structured basis for future feasibility, implementation, and effectiveness evaluations. However, its clinical effectiveness, safety, feasibility, and external validity remain to be established.

## 1. Introduction

Postpartum urinary retention (PUR) is commonly defined as the inability to void spontaneously within a specified period after delivery or catheter removal, or the presence of significant post-void residual urine volume despite spontaneous voiding [1]. PUR is one of the most common urinary complications following childbirth and represents an important but frequently under-recognized maternal health concern. Previous studies have reported substantial variations in the incidence of PUR, ranging from 0.18% to 47%, which may be attributable to differences in diagnostic criteria, population characteristics, and clinical management practices [2,3]. In China, the incidence of PUR has been reported to be approximately 10% and has shown an increasing trend in recent years [4]. Multiple obstetric factors, including instrumental delivery, prolonged labor, episiotomy, perineal trauma, epidural analgesia, oxytocin augmentation, and induction of labor, have been identified as potential risk factors for PUR [2,3,5,6]. Failure to identify and manage PUR in a timely manner may lead to short- and long-term adverse maternal outcomes. Acute bladder overdistension can impair detrusor muscle function and damage bladder sensory pathways, resulting in persistent voiding dysfunction. In severe cases, excessive bladder distension may contribute to increased postpartum bleeding, urinary tract infection, and, rarely, bladder rupture [7,8]. Furthermore, prolonged postpartum voiding dysfunction may negatively affect maternal quality of life and pelvic floor recovery [9]. Therefore, effective bladder management during the perinatal period is essential for preventing bladder overdistension, promoting early recognition of dysfunction, and reducing complications.

Several international organizations have provided recommendations related to maternal bladder care. For example, clinical guidelines have emphasized the importance of monitoring bladder function, encouraging regular voiding, assessing postpartum urinary function, and appropriately managing women at increased risk of urinary retention. However, existing recommendations remain heterogeneous, with considerable variation in screening approaches, timing of assessment, catheter management, and criteria for intervention [7]. In clinical practice, bladder management is often fragmented and highly dependent on individual healthcare professionals’ experience rather than standardized evidence-based pathways. This variability highlights the need for a structured and context-specific intervention that integrates prevention, early identification, and management of bladder dysfunction throughout the perinatal period. Perinatal bladder management represents a complex healthcare problem involving multiple interacting factors, including physiological changes during pregnancy, labor-related trauma, analgesia-related sensory impairment, healthcare provider behaviors, and maternal knowledge and self-management abilities. Therefore, a single intervention is unlikely to adequately address this multifactorial issue. Complex interventions require systematic development approaches that consider theoretical mechanisms, available evidence, stakeholder perspectives, and implementation feasibility. The Medical Research Council (MRC) framework for developing and evaluating complex interventions provides a methodological approach for designing interventions with multiple interacting components. The updated MRC framework emphasizes the importance of identifying intervention mechanisms, incorporating theory, engaging stakeholders, and developing interventions that are appropriate for specific healthcare contexts [10]. Applying this framework may facilitate the translation of evidence into a practical and implementable perinatal bladder management intervention.

Although existing studies have explored individual strategies for preventing or treating PUR, such as bladder assessment, catheterization protocols, and non-pharmacological approaches, comprehensive interventions integrating antenatal preparation, intrapartum monitoring, postpartum management, and urinary retention treatment remain limited, particularly within the Chinese healthcare context. Therefore, this study aimed to develop a theory-informed, evidence-based intervention for perinatal bladder management among pregnant women, guided by the MRC framework for complex interventions.

## 2. Materials and Methods

### 2.1. Study Design and Framework

This study was designed as an intervention development study guided by the MRC framework for developing and evaluating complex interventions. The updated MRC framework emphasizes the importance of integrating theory, evidence, stakeholder perspectives, and iterative refinement during the development of complex healthcare interventions. This study focused on the development phase of the MRC framework and aimed to establish an evidence-based and context-specific perinatal bladder management intervention for pregnant women.

**Figure 1:**
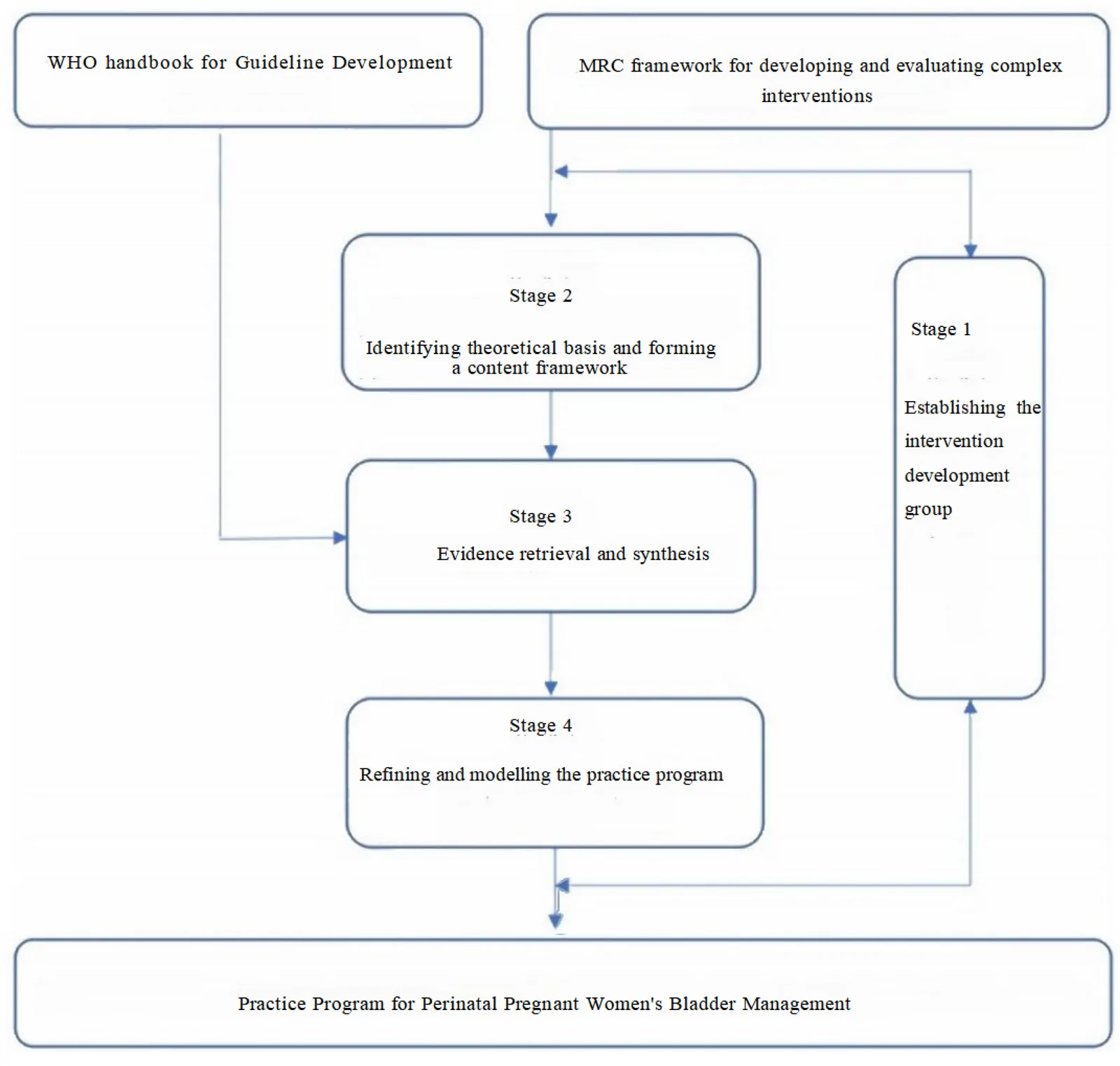
Development process of the perinatal bladder management intervention protocol.

The development process consisted of four sequential stages: (1) establishment of a multidisciplinary intervention development group; (2) identification of the theoretical foundations and development of the intervention framework; (3) systematic identification, appraisal, and synthesis of evidence; and (4) expert-based refinement and consensus assessment of the intervention components (Figure 1). Figure 1 illustrates the overall development process of the intervention.

### 2.2. Stage 1: Establishment of the Multidisciplinary Intervention Development Group

A multidisciplinary intervention development group was established to ensure that multiple perspectives relevant to perinatal bladder management were incorporated throughout the development process. The group included professionals from obstetrics, anesthesiology, midwifery, nursing management, evidence-based practice, and maternal representation. The development group was responsible for determining the intervention framework, reviewing and integrating evidence, discussing the feasibility of intervention components, and developing the preliminary perinatal bladder management intervention.

### 2.3. Stage 2: Identification of Theoretical Foundations and Development of the Intervention Framework

The theoretical foundation of the intervention was established through two complementary approaches: a review of the physiological mechanisms underlying postpartum voiding dysfunction and a qualitative study guided by the Theoretical Domains Framework (TDF). First, the physiological mechanisms associated with PUR were reviewed to identify potential intervention targets, including prevention of bladder overdistension, preservation of bladder sensory function, and promotion of effective voiding. Second, a descriptive qualitative study guided by the TDF was conducted to explore behavioral and contextual determinants of perinatal bladder management among women following vaginal delivery and healthcare professionals. Purposive sampling was used to recruit 16 women following vaginal delivery and 18 healthcare professionals. Semi-structured interviews were conducted to explore participants’ experiences and perceptions, as well as the barriers to and facilitators of perinatal bladder management. Before each interview, written informed consent was obtained from all participants. Participants were assured that participation was voluntary, that they could withdraw at any time, and that their responses would remain confidential and anonymous. The interview guide was developed based on the TDF and relevant literature, with questions tailored to the characteristics of different participant groups. The interviewers had received qualitative research training. Interviews with women were conducted 6–10 h after vaginal delivery in a quiet and private setting. Each participant completed one interview lasting approximately 40–50 min. Interviews were audio-recorded and accompanied by field notes documenting non-verbal information and contextual observations. Audio recordings were transcribed within 24 h, and ambiguous statements were clarified with participants when necessary. The analysis followed Colaizzi’s seven-step procedure, including repeated reading of transcripts and field notes, extraction of significant statements, formulation of meaning units and initial codes, clustering of related codes into subthemes and themes, development of a comprehensive description and underlying structure, and validation of the preliminary findings with selected participants. Discrepancies were resolved through discussion, with a third researcher consulted when necessary. Before formal interviews and coding, researchers documented and bracketed their preconceptions regarding perinatal bladder management. Reflexive journals and field notes were maintained throughout the study. Double coding, team discussion, peer debriefing, and negative case analysis were used to enhance the credibility and dependability of the qualitative findings. Sample size was determined by data saturation, with recruitment and analysis conducted iteratively until no new substantive themes emerged. The qualitative findings were mapped onto the relevant TDF domains to identify behavioral and contextual determinants of perinatal bladder management. The findings from the physiological review and qualitative study were then integrated by the multidisciplinary intervention development group to identify key intervention targets and establish the preliminary intervention framework. Women who underwent cesarean delivery were not included in the qualitative interviews because the qualitative study focused on experiences of vaginal delivery. This limits the applicability of the findings to women undergoing cesarean delivery.

### 2.4. Stage 3: Evidence Identification and Synthesis

Evidence synthesis was conducted to identify the best available evidence supporting each intervention component. The evidence retrieval process followed the “5S” model of evidence-based healthcare resources, which organises evidence from the highest to the lowest level: Systems, Summaries, Synopses, Syntheses, and Studies. In this study, the 5S model was operationalised as a top-down hierarchical search and evidence-selection framework. Formal grades of recommendation (strong/weak) were not assigned. The appropriateness of several components depends on the clinical context and individualized assessment, and the evidence base for some components remains uncertain or is based on expert consensus. JBI levels of evidence were reported where available, and conditional language was used for components with safety or implementation uncertainties.

#### 2.4.1. Operationalisation of the 5S Model

The 5S hierarchy was operationalised as follows. At the Systems and Summaries levels, we searched clinical decision-support resources, guideline databases, and evidence summary databases, including the JBI EBP Database, Guidelines International Network, Royal College of Nursing (UK), Royal College of Obstetricians and Gynaecologists (Ireland), Queensland Health, SA Health, and the International Continence Society. At the Synopses/Syntheses level, we searched Cochrane Library, PubMed, Embase, Web of Science, and CINAHL for synopses of syntheses, systematic reviews, and meta-analyses. At the Studies level, we searched PubMed, Embase, Web of Science, CNKI, Wanfang Database, and SinoMed for randomised controlled trials and other original studies. Citation tracking was also performed to identify additional relevant sources.

The search was conducted from database inception to 14 February 2025. Chinese and English databases were searched using combinations of keywords related to perinatal women, PUR, bladder management, urinary catheterisation, pelvic floor exercise, and related interventions. The search was performed sequentially from the highest to the lowest 5S level. If a current, high-quality, and directly applicable guideline, evidence summary, or systematic review was available for a given topic, it was prioritised as the primary evidence source. Lower-level evidence was consulted only when higher-level evidence was absent, outdated, not directly applicable to the Chinese perinatal context, or insufficient to address a specific intervention component. Expert consensus statements were treated as supplementary summary-level evidence when higher-level evidence was unavailable or when contextual or operational guidance was needed.

Two researchers independently screened titles, abstracts, and full texts according to the prespecified eligibility criteria. Disagreements were resolved through discussion, and a methodological expert was consulted when consensus could not be reached. Data extracted from included sources included the evidence source, publication year, type of evidence, target population, intervention content, and evidence level. The extracted recommendations were then mapped to the four core domains of the intervention: antenatal bladder management, intrapartum bladder management, postpartum bladder management, and management of urinary retention. Health education was treated as a cross-cutting theme and integrated into these domains. When multiple sources addressed the same topic, recommendations were prioritised according to evidence level, methodological quality, recency, and applicability to the study context. Conflicting recommendations were resolved through multidisciplinary group discussion. This process resulted in 34 evidence-based recommendations, which were subsequently refined into the final 29 intervention components through Delphi consultation.

#### 2.4.2. Search Strategy

A comprehensive literature search was conducted from database inception to 14 February 2025. Chinese and English databases were searched using combinations of keywords related to perinatal women, postpartum urinary retention (PUR), bladder management, urinary catheterization, pelvic floor exercise, and related interventions.

#### 2.4.3. Eligibility Criteria

Studies were eligible if they met the following criteria: (1) guidelines, evidence summaries, systematic reviews, expert consensus statements, or high-quality original studies available from electronic databases; (2) focused on prevention, assessment, management, or nursing care related to perinatal bladder dysfunction or PUR; (3) included pregnant women or postpartum women as study populations; and (4) represented the most recent version when updated documents were available.

Studies were excluded if they were duplicates, lacked sufficient methodological information, were unavailable in full text, or were published in languages other than Chinese or English.

#### 2.4.4. Quality Appraisal and Evidence Integration

Methodological quality assessment was independently performed by two researchers. Clinical guidelines were appraised using the Appraisal of Guidelines for Research and Evaluation II (AGREE II) instrument [11]; systematic reviews were evaluated using AMSTAR 2 [12]; expert consensus statements were assessed using the JBI Critical Appraisal Checklist for Text and Opinion Papers (2020) [13]; evidence summaries were assessed using the appropriate evidence summary appraisal criteria [14]; and randomized controlled trials were evaluated using the Cochrane Risk of Bias 2 (RoB 2) tool [15]. Any disagreements between reviewers were resolved through discussion. If consensus could not be reached, a methodological expert was consulted. Each recommendation was graded using the JBI Levels of Evidence (Level 1–5). Level 1 refers to experimental designs, including randomized controlled trials and systematic reviews of randomized controlled trials. Level 2 refers to quasi-experimental designs. Level 3 refers to observational-analytic designs, including cohort and case–control studies. Level 4 refers to observational-descriptive designs, including case series and cross-sectional studies. Level 5 refers to expert opinion, bench research, or guideline-based recommendations without directly cited research evidence. For guidelines and expert consensus documents, the underlying evidence level was reported where available; when no specific level was reported, the recommendation was labelled as guideline-based or expert consensus (Level 5). The evidence summary follows these principles: ① If the contents are consistent, select the evidence that is concise, easy to understand and conforms to professional expression; ② if the contents are complementary, combine them based on logical language to form evidence; and ③ if the contents conflict, prioritize evidence based on evidence from reliable sources, high-quality evidence, and the latest authoritative literature. In summary, if there is still evidence conflict, consult experts in evidence-based research for decision-making.

### 2.5. Stage 4: Expert-Based Refinement and Consensus Assessment of the Intervention

Following evidence synthesis, a preliminary intervention framework was developed and reviewed using a Delphi expert consultation process. The Delphi method has been widely used to develop nursing and rehabilitation programmes, and its utility has been reported previously [16].

#### 2.5.1. Expert Selection

Experts were recruited based on the following predefined inclusion criteria: (1) professional expertise in obstetrics, midwifery, or maternal nursing; (2) a bachelor’s degree or higher; (3) an intermediate professional title or higher; and (4) more than 10 years of clinical experience in a related field. Members of the intervention development group were excluded from the Delphi panel to maintain independence during the consensus process. All experts provided written informed consent before participating in the Delphi consultation. The Delphi panel was composed mainly of nursing and midwifery professionals from tertiary hospitals in a limited number of Chinese cities, which may limit the representativeness of the panel.

#### 2.5.2. Delphi Consultation Process

Two rounds of Delphi consultation were conducted with 18 experts. A structured questionnaire was developed based on the preliminary intervention framework derived from the evidence synthesis and the TDF-guided qualitative study. Each item was rated for importance and feasibility using a 5-point Likert scale. Importance was defined as the extent to which the item was considered clinically necessary and prioritized for preventing PUR, promoting early identification of bladder dysfunction, and reducing maternal complications. Feasibility was defined as the extent to which the item could be safely implemented in routine Chinese perinatal practice, considering resource availability, workload, staff skills, infection-control requirements, and acceptability to women and clinicians. The anchors were: importance, 1 = very unimportant to 5 = very important; feasibility, 1 = very infeasible to 5 = very feasible. The coefficient of variation (CV) was calculated as SD/mean for each item. Before the first round, the following a priori item-level consensus and decision criteria were specified and documented in the Delphi protocol: Retention: importance mean ≥3.5 and CV ≤ 0.25; feasibility mean ≥3.5 and CV ≤ 0.25; and no substantial objection from experts. Modification: items not meeting retention criteria but with importance mean ≥3.5 and CV 0.26–0.30; or feasibility mean 3.0–3.49 or CV 0.26–0.30; or specific expert comments indicating a need for clarification, rewording, or operational adjustment. Deletion: Importance mean <3.5 or CV > 0.30; feasibility mean <3.0 or CV > 0.30; or repeated substantial objections that could not be resolved through revision. Decision sequence: Retention criteria were applied first. Items meeting retention criteria were retained. Items not meeting retention criteria were then evaluated against modification criteria. Items meeting neither retention nor modification criteria were deleted. In the first round, experts also rated their judgment basis (Ca) and familiarity (Cs) with the content areas, and the expert authority coefficient (Cr) was calculated as Cr = (Ca + Cs)/2. After each round, the development group summarized the quantitative results and qualitative comments. The anonymous results were fed back to the experts together with the revised questionnaire. Experts could re-rate items and provide further comments. Consensus was considered achieved when all retained items met the retention criteria and no new substantive modifications were proposed. Kendall’s W was used as a panel-level indicator of ranking agreement and was not used as the sole criterion for retaining, modifying, or deleting individual items. Item-level decisions were based on the predefined importance and CV criteria.

### 2.6. Ethical Considerations

This study was approved by the Ethics Review Committee of Peking University People’s Hospital (2025PHB186-001). All participants provided written informed consent before participation. For the qualitative interviews, postpartum women and healthcare professionals were informed of the study purpose, interview procedures, voluntary participation, the right to withdraw at any time without any effect on their care or employment, and the measures taken to ensure anonymity and confidentiality. Written informed consent was obtained before each interview. For the Delphi consultation, experts were informed of the study purpose, the consultation process, and their right to withdraw, and written informed consent was obtained before the first round. All data were anonymised and used only for research purposes.

### 2.7. Safety and Implementation Appraisal of Recommendations

Before finalising the protocol, each recommendation was appraised for potential safety and implementation implications by the multidisciplinary development group. The following questions were considered: (1) Does the recommendation involve an invasive procedure, medication, device, or technique with potential adverse effects? (2) Are there contraindications, precautions, or resource requirements? (3) Can the recommendation be delivered safely by the existing workforce in the Chinese perinatal context? (4) What mitigations are needed to reduce risk or improve feasibility? (5) Should the recommendation be routine, conditional, optional, or protocol-dependent?

Recommendations with potential safety or implementation concerns were reworded as conditional, optional, or protocol-dependent, and were retained only when the expected benefits outweighed the risks and the intervention was considered feasible in the Chinese perinatal context.

## 3. Results

### 3.1. Stage 1 Result: Establishment of the Intervention Development Team

The intervention development team established for this study consisted of nine members: two obstetricians, one anesthesiologist, two midwives, one obstetric nurse, one nursing management expert, one evidence-based practice expert, and one maternal representative. The team’s main tasks were to (1) determine the content framework for the perinatal bladder management protocol; (2) collect, appraise, and synthesize relevant evidence for each topic; and (3) develop and revise the protocol.

### 3.2. Stage 2 Results: Theoretical Foundations and Preliminary Intervention Framework

The intervention framework was informed by the pathophysiological mechanisms underlying postpartum voiding dysfunction. During the delivery process, the continuous compression of the fetal presenting part on the bladder neck and pelvic floor nerves can cause bladder mucosal edema and temporary impairment of nerve terminal conduction function, which may impair transmission of bladder-filling signals [17]. Epidural analgesia may also reduce detrusor contractility through neural blockade [7]. If the bladder is overfilled without timely intervention, it can lead to irreversible stretching damage of the detrusor muscle. Antenatal pelvic floor muscle exercises aim to establish urinary function reserves [18]; during labour, fluid intake/output monitoring and regular voiding are intended to prevent excessive bladder filling [17]. Early postpartum voiding and allowing women to choose a comfortable voiding position may facilitate voiding [19]. Risk-stratified assessment within 4–6 h after delivery may help identify women who require intervention [7]. These mechanisms supported the inclusion of regular voiding, fluid and urine output monitoring, early postpartum voiding assessment, and timely risk-stratified intervention in the preliminary framework.

The TDF-guided qualitative study [20] identified three themes that facilitated perinatal bladder management and three themes that hindered it. The facilitating themes included: (1) cognition and professional support promoting proactive voiding; (2) behavioral adaptation and maintenance of behavioral intention; and (3) risk identification, monitoring processes, and organizational resource support. The barrier themes included: (1) physical discomfort, treatment-related factors, and environmental constraints limiting behavioral implementation; (2) insufficient knowledge, urinary-urge awareness, and risk appraisal; and (3) reduced priority of bladder management, inadequate social support, and discontinuity of the management process.

These themes were mapped onto relevant TDF domains, including knowledge, skills, beliefs about capabilities, beliefs about consequences, motivation and goals, social influences, environmental context and resources, memory, attention and decision processes, behavioral regulation, and the nature of the behavior.

The qualitative findings were then translated into intervention targets. Insufficient knowledge and urinary-urge awareness supported the inclusion of structured maternal education and voiding assessment; physical discomfort and environmental constraints informed supportive voiding measures; and identified gaps in risk recognition and care continuity supported standardized risk assessment, monitoring, documentation, and feedback processes.

Based on the physiological evidence and TDF-guided qualitative findings, the preliminary intervention framework comprised four core domains: antenatal bladder management, intrapartum bladder management, postpartum bladder management, and management of urinary retention. Health education was considered a cross-cutting component rather than a separate intervention domain and was integrated into the four core domains during subsequent intervention development.

### 3.3. Stage 3 Results: Evidence Synthesis

**Figure 2:**
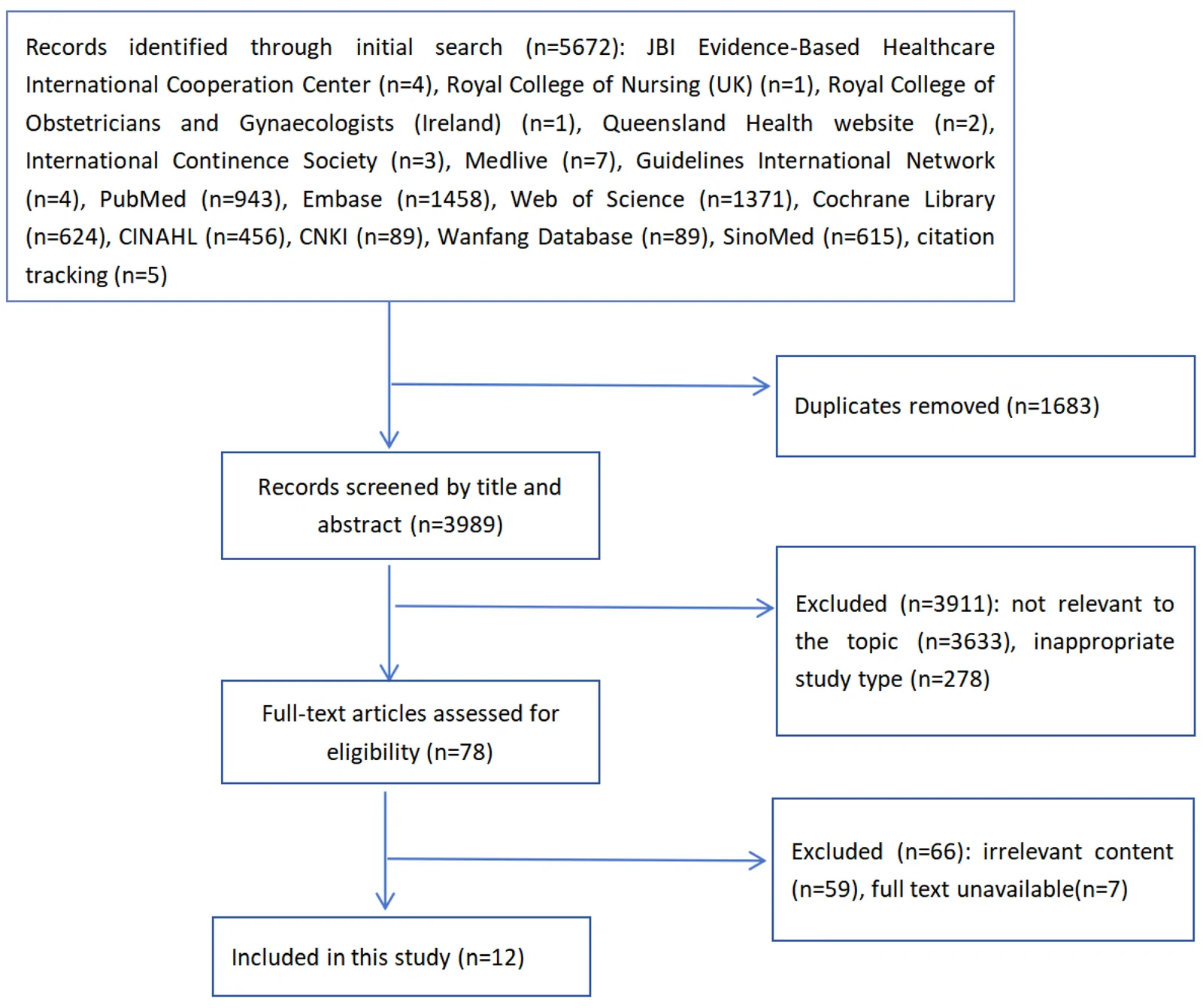
Literature screening flowchart.

This study initially retrieved 5672 documents, of which 1683 were duplicates. Literature search strategy and results is provided in Supplementary Table S1. After reading the titles and abstracts of the remaining 3989 documents, 3911 were excluded. After reading the full texts and conducting quality evaluations, 66 were excluded, leaving 12 documents in total, including four guidelines [21,22,23,24], three evidence summaries [25,26,27], three expert consensus documents [18,28,29], one systematic review of guidelines [30], and one randomized controlled trial [31]. The literature-screening process is shown in Figure 2. The general characteristics of the included literature are summarized in Table 1. Four guidelines were included, and for each of these guidelines, the number of domains with ≥30% coverage was ≥3, and they were rated as grade B. Three expert consensus documents were included. For each document, item 6 was rated “unclear,” whereas the remaining items were rated “yes.” Three evidence summaries from JBI were included. For all three summaries, items 3 and 4 were rated “partially,” item 8 was rated “no,” and the remaining items were rated “yes.” One randomized controlled trial was included. Items 5 and 6 were rated as “unclear,” whereas all other items were judged to indicate a low risk of bias. 1 systematic review of guidelines was included, and 4 original guidelines were traced for evaluation. One guideline was rated grade A, two were rated grade B, and one was rated grade C. After excluding the content of the C-level guidelines, the overall quality was good and it was included. A total of 34 evidence-based recommendations were synthesized from the literature on perinatal bladder management. They were organized into four domains: antenatal bladder management, intrapartum bladder management, postpartum bladder management, and management of urinary retention. Health education was treated as a cross-cutting theme and integrated into the relevant domains. Health education was treated as a cross-cutting theme rather than a fifth domain; these recommendations were integrated into the relevant domains, particularly antenatal education, pelvic floor muscle training education, infection prevention, and discharge catheter care. The specific content of the evidence is shown in Table 2.

### 3.4. Stage 4 Results: Finalisation of the Protocol

Based on the established content framework and the results of the evidence review, a draft bladder management protocol was developed. Relevant experts were invited to revise the draft, drawing on their extensive clinical experience and in-depth theoretical knowledge.

#### 3.4.1. Expert Consultation

Using the preliminary protocol developed from the evidence summary and expert meetings, the Delphi method was employed in this study to refine the protocol. A total of 18 experts from 14 Grade III Class A hospitals in Beijing, Shanghai, Hangzhou, and Qingdao were invited to participate in two rounds of consultation. Among them, 5 were aged 30–39 years, 10 were aged 40–49 years, and 3 were aged 50–59 years; one expert held a doctoral degree, seven held a master’s degree, and ten held a bachelor’s degree; 94.44% held senior or associate senior professional titles. The inclusion criteria for experts were: (1) engaged in nursing management, clinical obstetric nursing, or midwifery; (2) holding at least a mid-level professional title; (3) having more than 10 years of working experience in the field; and (4) willing to participate with informed consent. Most experts were from tertiary-level A hospitals in Beijing, Shanghai, Hangzhou, and Qingdao, and the panel was predominantly composed of nursing and midwifery professionals. This composition should be considered when interpreting the applicability of the findings to other settings. Members of the intervention development group did not participate in the expert consultation. The response rate for both rounds was 100%, and in the first round, 13 experts provided written comments, indicating a high level of engagement. In the first round, the expert authority coefficient (Cr) was 0.956, with a judgment basis coefficient (Ca) of 0.944 and a familiarity coefficient (Cs) of 0.967. In the second round, Cr was 0.972, with Ca = 0.956 and Cs = 0.989, demonstrating a very high level of expert authority. In the first round, the coefficient of variation (CV) for importance scores of all items ranged from 0 to 0.33, Kendall’s W was 0.198 (*p* < 0.001), and the chi-square value was 106.866. In the second round, CV ranged from 0 to 0.17, Kendall’s W was 0.120 (*p* < 0.001), and the chi-square value was 60.499. These Kendall’s W values indicate variability in expert rankings and should be interpreted cautiously; possible contributing factors are considered in the Discussion.

#### 3.4.2. Formation of the Protocol

After the first round of expert consultation, one indicator was deleted and 13 were revised. For example, for item 1.1, experts suggested changing “history of previous voiding difficulties, urinary incontinence, bladder injury, urinary retention, and constipation” to “history of voiding and defecation abnormalities and surgical history,” and clarifying the time point for obtaining relevant maternal medical history and general information. After the second round, one duplicated indicator was deleted and four were revised, such as changing “bladder sensation” (in item 1.2) to “bladder filling sensation.” Based on the experts’ suggestions and the detailed comments provided, the development group subsequently held a dedicated meeting to discuss the specific application and further refine the draft. Final consensus was reached, and the expert recommendations were integrated into the initial protocol, resulting in the final perinatal bladder management protocol (Table 3).

## 4. Discussion

### 4.1. Principal Findings

This study developed a theory-informed and evidence-based perinatal bladder management intervention for pregnant women using the MRC framework for complex intervention development. Through a structured process integrating evidence synthesis, qualitative exploration based on the Theoretical Domains Framework (TDF), multidisciplinary collaboration, and Delphi consultation, a comprehensive intervention framework comprising four domains and 29 components was developed.

Unlike interventions focusing solely on the treatment of PUR, the developed intervention adopts a continuum-of-care approach covering antenatal preparation, intrapartum monitoring, postpartum assessment, and urinary retention management. This comprehensive approach reflects the multifactorial nature of perinatal bladder dysfunction and provides a structured pathway for prevention, early identification, and management.

### 4.2. Comparison with Previous Studies

Previous studies have primarily focused on individual aspects of PUR management, such as identifying risk factors, assessing postpartum residual urine volume, or implementing catheterization protocols. For example, two recent systematic reviews and meta-analyses have identified a range of risk factors, including instrumental delivery, epidural analgesia, perineal injury, labor augmentation, and prolonged labor duration [2,3]. Although these strategies provide important evidence for clinical decision-making, they often address isolated stages of bladder dysfunction management rather than the entire perinatal care process.

Existing clinical guidelines and consensus statements have provided recommendations regarding postpartum bladder assessment and management; however, considerable heterogeneity remains regarding screening timing, assessment methods, and intervention thresholds [7]. A national survey of 295 academic institutions in the United States found that 56.3% of hospitals lacked a PUR protocol, and among those with protocols, wide variations existed in time limits, residual volume thresholds, and catheterization criteria [32]. This variability may contribute to inconsistent clinical practices and reliance on individual healthcare professionals’ experiences. A scoping review by De Rosa et al. [33] systematically examined risk factors and management interventions for PUR, concluding that prevention through prompt recognition of risk factors and development of standardized protocols is essential for improving clinical practice. Similarly, an expert review emphasized that nationally accepted definitions and management algorithms for PUR are lacking, and development of such guidelines is essential for both patient care and research design [27]. The intervention developed in this study extends previous work by integrating preventive and management strategies across the perinatal period. By incorporating antenatal education, intrapartum bladder monitoring, postpartum assessment, and targeted management of urinary retention, the intervention provides a more comprehensive framework that may better support continuity of maternal care.

### 4.3. Value of the MRC Framework and Theory-Informed Development Approach

A major strength of this study is the application of the MRC framework to guide intervention development. Complex healthcare problems are influenced by multiple interacting biological, behavioral, organizational, and contextual factors; therefore, interventions developed without a systematic framework may lack theoretical coherence and implementation feasibility. The MRC framework emphasizes understanding intervention mechanisms, integrating evidence, engaging stakeholders, and considering implementation contexts [10]. In this study, physiological evidence was combined with behavioral determinants identified through TDF-guided qualitative interviews, allowing the intervention components to address not only clinical requirements but also behavioral, interpersonal, and organizational determinants of perinatal bladder management.

The use of the TDF provided a behavioral and contextual basis for the intervention components. The qualitative findings indicated that knowledge, awareness of urinary urge, motivation, emotional and physical discomfort, social support, environmental conditions, and the organization of monitoring and care processes could influence bladder management behaviors [34]. These findings helped translate the qualitative evidence into specific intervention targets, including maternal education, timely voiding support, risk identification, standardized monitoring, and continuity of care. This theory-informed approach may enhance the relevance and acceptability of the intervention because it considers both clinical evidence and real-world implementation challenges.

### 4.4. Clinical Implications and Implementation Considerations

The developed intervention has potential implications for improving the standardization of perinatal bladder management. Previous evidence has demonstrated that implementation of clinical practice guidelines for PUR can decrease variance in clinical practice, reduce PUR rates, and decrease the number of urinary catheterizations [9]. A universal postpartum voiding protocol has also been shown to be associated with a low incidence of PUR, and awareness of risk factors combined with implementation of standardized voiding protocols may facilitate early identification and prevention [35]. By providing structured recommendations across different stages of maternity care, the intervention may assist healthcare professionals in identifying women at risk, implementing timely assessments, and delivering consistent bladder care.

However, successful implementation requires consideration of local healthcare contexts. National surveys have revealed that a substantial proportion of institutions lack PUR protocols. One study found that only 30.3% of obstetrics and gynecology residents reported that their institutions had a PUR prevention protocol [36]. Even among institutions with protocols, substantial variation remains in time limits, residual-volume thresholds, and catheterization criteria [32]. Furthermore, there are no universal diagnostic criteria for PUR, and institution-specific clinical guidelines vary greatly. Factors such as staffing resources, workflow organization, professional training, and institutional protocols may influence intervention adoption. Therefore, before widespread implementation, feasibility testing and pilot studies are required to evaluate acceptability, practicality, and resource requirements in different clinical settings.

Future studies should investigate the effectiveness of this intervention using rigorous evaluation designs, such as controlled clinical studies or implementation evaluations. Relevant outcomes may include the incidence of PUR, catheterization rates, maternal urinary function recovery, healthcare utilization, and patient-reported outcomes. Given that the accuracy of bladder scanners in the postpartum population requires further validation, future research should also consider the reliability of assessment tools used in protocol implementation. Additionally, long-term follow-up studies are needed to evaluate the impact of standardized bladder management on persistent voiding dysfunction and quality of life.

### 4.5. Strengths and Limitations

This study has several strengths. First, it followed a recognized international framework for complex intervention development, increasing methodological transparency and reproducibility. Second, the intervention was developed through integration of multiple evidence sources, theoretical analysis, and multidisciplinary expert input. Third, the involvement of healthcare professionals and postpartum women allowed the intervention to incorporate both clinical and experiential perspectives.

Several limitations should also be acknowledged. First, we did not assign formal grades of recommendation. While this may limit the certainty with which some components can be applied, it reflects the context-dependent nature of perinatal bladder management and the intention to position the protocol as a clinical reference framework rather than a prescriptive guideline. The intervention has not yet been evaluated in clinical practice; therefore, its effectiveness and cost-effectiveness remain unknown. Second, although multidisciplinary experts participated in Delphi consultation, the expert panel size was relatively limited and may not fully represent all clinical contexts. Third, the stakeholder composition may limit the applicability of the intervention. The qualitative interviews were conducted with women 6–10 h after vaginal delivery, and women who underwent cesarean delivery were not included. Because cesarean delivery is associated with different bladder management needs, including postoperative catheterization, mobility restrictions, and pain management, the intervention may require adaptation for this population. In addition, the Delphi panel consisted mainly of nursing and midwifery professionals from tertiary hospitals in a limited number of Chinese cities. Their perspectives may not fully represent those of clinicians in lower-resource institutions, primary care settings, or other regions. Healthcare systems also differ in staffing, catheterization practices, availability of bladder scanners, and medication protocols. Therefore, the intervention should be regarded as a context-specific protocol that requires adaptation and validation before implementation in different settings, including cesarean delivery populations, lower-resource institutions, other regions, and different healthcare systems. Additionally, the Delphi agreement, as indicated by Kendall’s W, was modest and decreased from 0.198 in the first round to 0.120 in the second round (*p* < 0.001 for both). Although these values were statistically significant, statistical significance indicates that the observed agreement was unlikely to be due to chance; it does not indicate strong consensus. Kendall’s W reflects the degree of agreement in expert rankings, and lower values indicate greater variability in how experts prioritized the intervention components. It is important to distinguish between different properties of the Delphi process. The response rate reflects participant engagement; the expert authority coefficient (Cr) reflects the panel’s level of expertise and familiarity with the content; and Kendall’s W reflects inter-expert agreement in ranking. These are separate properties, and a high Cr does not compensate for limited concordance. Therefore, the modest W values should be acknowledged as a limitation of the consensus process. One possible interpretation is that the multidisciplinary composition of the expert panel contributed to variability in prioritization; however, this study was not designed to formally test this hypothesis, and other unmeasured factors may also have contributed, such as differences in clinical experience, institutional contexts, and the dual assessment of importance and feasibility. The final items were retained based on the predefined importance and CV criteria rather than on Kendall’s W alone. Further validation in diverse settings is warranted. Some recommendations in the final protocol have potential safety or implementation implications, particularly those involving medication, acupuncture-related interventions, catheterization thresholds and durations, and bladder scanner use, more RCT studies will be needed in the future. However, the clarification of the Delphi decision criteria and the acknowledgement of the absence of clinical validation are positive developments. However, the outstanding clinical concerns are directly relevant to the scientific reliability and potential implementation of the proposed intervention.

### 4.6. Future Directions

Future research should focus on feasibility assessment, implementation evaluation, and effectiveness testing of the developed intervention. Pilot implementation studies should explore whether the intervention can be integrated into routine maternity care and identify potential barriers and facilitators. Subsequently, larger-scale studies are needed to determine whether the intervention can reduce the incidence of PUR, improve maternal urinary outcomes, and enhance the quality of perinatal care. Additionally, future validation studies should include women undergoing cesarean delivery, clinicians from lower-resource institutions and primary care settings, and stakeholders from other regions and healthcare systems to evaluate the transferability and effectiveness of the intervention in diverse contexts.

## 5. Conclusions

Using the MRC framework for complex intervention development, this study developed a theory-informed and evidence-based perinatal bladder management intervention for pregnant women. The intervention integrates evidence synthesis, behavioral theory, multidisciplinary expertise, and stakeholder perspectives to address the multifactorial nature of perinatal bladder dysfunction. Further feasibility and effectiveness evaluations are warranted before clinical implementation.

## Figures and Tables

**Table 1:** Characteristics of the included studies.

Included Literature	Year	Source	Type	Research Topic
RCPI [21]	2019	RCPI	Guideline	Management of urinary retention during pregnancy, the postpartum period, and after gynaecological surgery
Tucker et al. [22]	2018	SA Health	Guideline	Maternal bladder management during intrapartum and postpartum periods
RCN [23]	2024	RCN	Guideline	Bladder and bowel care during pregnancy
QCG [24]	2022	QCG	Guideline	Normal birth
BPsychSc et al. [25]	2023	JBI	Evidence summary	Intrapartum bladder management
BPsychSc et al. [26]	2023	JBI	Evidence summary	Postpartum bladder management
Othman et al. [27]	2021	JBI	Evidence summary	Intrapartum and postpartum bladder management
Nutaitis A C et al. [28]	2023	PubMed	Expert consensus	PUR management
Yang Z. et al. [29]	2020	CNKI	Expert consensus	Clinical application of carboprost methylate
Sun X.L. et al. [18]	2024	CNKI	Expert consensus	Prevention of pelvic floor dysfunction during pregnancy
Divakova et al. [30]	2022	PubMed	Systematic review	Postpartum bladder care
Yuhang et al. [31]	2022	PubMed	Randomized controlled trial	Effect of acupoint hot compress on PUR

Note: RCPI: Royal College of Physicians of Ireland; RCN: Royal College of Nursing; QCG: Queensland Clinical Guidelines; CNKI: China National Knowledge Infrastructure; JBI: Joanna Briggs Institute.

**Table 2:** Evidence summary of bladder management for pregnant women.

Topic	Evidence Content	Level of Evidence
Antenatal bladder management	1. Obtain relevant medical history and general information, including: urinary symptoms, fluid intake, time since last normal void, history of previous voiding difficulties, urinary incontinence, bladder injury, urinary retention, and constipation [21,25].	5
	2. Encourage pelvic floor muscle training. Recommend 15 min of daily practice, starting at 28 weeks of gestation and continuing for at least 6 weeks [18,23].	5
	3. Pelvic floor muscle training should initially be performed in a supine or sitting position, then gradually transition to upright and active positions. Two types of exercises are recommended to train both fast-twitch and slow-twitch muscles: (a) sustained contractions (hold as long as possible, generally 6–8 s) and (b) quick contractions with rapid release. Avoid breath-holding during exercises [18,23].	5
	4. Encourage pregnant women to drink 1.5–2 L of fluid daily during pregnancy. Limit caffeine and carbonated beverages, and avoid alcohol [23].	5
Intrapartum bladder management	5. During labour, complete a fluid balance chart recording all oral and intravenous fluids and voiding volumes, and note the use of oxytocin [21,27].	5
	6. Encourage voiding every 2 h and document each void [22,24].	5
	7. If the woman cannot void every 2 h during labour, consider intermittent or indwelling urinary catheterisation until the onset of the second stage [21,22].	5
	8. For women using intermittent catheterisation, the recommended frequency is at least every 4 h, with a target of less than 500 mL per catheterisation. If the drained volume exceeds 500 mL, the catheterisation interval should be shortened [25,26].	5
	9. Before entering the second stage of labour, any indwelling urinary catheter must be removed to avoid urethral injury [21,22].	5
	10. If neuraxial labour analgesia has been used, healthcare providers should assess whether the woman’s sensation of bladder filling is diminished or lost [25].	5
	11. If epidural analgesia has been used, consider leaving an indwelling catheter in place for 6 h after discontinuation of anaesthesia following vaginal delivery. In cases of severe perineal trauma (including obstetric anal sphincter injury), instrumental delivery, or manual removal of placenta, consider catheterisation for 12 h or longer, depending on the degree of perineal or vulval oedema and the woman’s pain level [21,22].	5
Postpartum bladder management	12. Advise women to start pelvic floor muscle training (e.g., Kegel exercises) as soon as possible after delivery, according to their individual condition, and to progress gradually to strengthen pelvic floor function [23].	5
	13. After delivery, encourage the woman to void. If she has not voided before leaving the delivery room, inform the postpartum ward staff so that the time and volume of the first void can be accurately recorded [21].	5
	14. After catheter removal, healthcare providers should assess and document the woman’s voiding attempts, sensation of voiding, and bladder function [26].	5
	15. Record the first two voids after delivery or after catheter removal for all women. Women should measure their voided volumes and report them to staff [21,27].	5
	16. Ensure that adequate pain relief and mobility are in place before the first void/catheter removal [26].	5
	17. Actively encourage all women to void every 2 h after delivery or after catheter removal [22,26,27].	5
	18. Routine non-pharmacological voiding methods include warm baths, running water sounds, hot compresses and massage over the bladder area, perineal irrigation, and acupuncture at relevant points. Pharmacological methods include intramuscular injection of neostigmine [30].	3
	19. Carboprost methylate suppositories promote contraction of gastrointestinal and bladder smooth muscle, thus facilitating postpartum voiding and intestinal gas expulsion, and can prevent and treat PUR and accelerate overall recovery. For this purpose, the recommended regimen is 1 mg administered vaginally, rectally, or sublingually immediately after fetal delivery, and again on the first and second postpartum days [29].	5
	20. Acupoint hot compress after epidural anaesthesia for vaginal delivery can reduce the incidence of PUR. The hot compress (45 °C) should be applied to: Shenque (RN8, centre of the umbilicus); Baliao points (BL31–BL34, the sacral foramina between the posterior superior iliac spine and the posterior midline); and Yongquan (KI1, depression on the sole of the foot when the foot is flexed) [31].	1
	21. The first void should occur within 4 to 6 h after delivery. If the woman has not voided within 4 h after the last recorded void or after catheter removal, initiate 2 h of conservative measures (e.g., adequate analgesia, fluid replacement, privacy) and start strict fluid balance charting. If signs of acute urinary retention appear within 4 h, intervention should not be delayed for an additional 2 h [21,22].	4
	22. Ensure adequate pain relief, inspect the perineum for any swelling, and recommend cold compresses [21].	4
	23. Ensure adequate fluid intake (1.5–2 L per day) [22].	5
	24. If conservative measures fail within 6 h after delivery, assess the woman for urinary retention and dehydration. Measure post-void residual volume using a urinary catheter or real-time bladder scanner within 10 min after voiding. Residual volume >150 mL is considered urinary retention [21,22].	5
	25. Report any bladder overdistension >600 mL to the senior clinician [22].	5
	26. If the woman has two spontaneous voids of 200 mL or more each, and has normal bladder sensation, no further action is required [21,27].	5
Management of urinary retention	27. A bladder scanner or urinary catheter (indwelling or in-and-out) can be used to assess complete bladder emptying. Bladder scanners accurately measure post-void residual volume after vaginal delivery; if available, a bladder scanner should be preferred over indwelling catheterisation as the first choice [21,23,27].	5
	28. Perform a routine urine test. If nitrites are positive, send for urine culture and sensitivity. Await results and review with the medical team for possible antibiotic treatment [21,22].	5
	29. If 150 mL ≤ catheterised volume < 500 mL, leave an indwelling urinary catheter for continuous drainage for 24 h. If catheterised volume is ≥500 mL but <1000 mL, leave the catheter for continuous drainage for 48 h. If catheterised volume is ≥1000 mL, leave the catheter for continuous drainage for 3–5 days [21].	5
	30. Inform the senior obstetrician, nurse in charge, and midwife of the acute episode of urinary retention, and involve them in ongoing care [21].	5
	31. Catheters should be removed in the morning, preferably after 6 a.m. If removal is expected after 6 p.m., it is recommended to leave the catheter in place until the next morning to facilitate measurement of the subsequent two voids [21].	5
Cross-cutting health education theme (integrated into the four core domains)	32. If the woman is to be discharged with a catheter in place, clearly specify the date for return for a trial without catheter (TWOC), and ensure she has received adequate education on bladder management and catheter care, including fluid intake, avoidance of constipation, perineal hygiene, hand hygiene, and catheter care [26,27].	5
	33. During antenatal care, all pregnant women should be educated about bladder care and pelvic floor muscle training. Healthcare professionals should not assume that women understand their own anatomy and physiology or know how to start pelvic floor exercises [23].	5
	34. Regularly educate pregnant women about infection prevention and the importance of understanding bladder function changes during pregnancy and childbirth. Do not assume that all women understand good care/hygiene practices, e.g., how to clean themselves after voiding and defecation [23].	5

Note: Health education recommendations are presented as a cross-cutting theme and are integrated into the four core domains rather than being counted as a separate fifth domain.

**Table 3:** Perinatal maternal bladder management protocol.

Item Content	Importance Score (Mean ± SD)	Coefficient of Variation
1. Antenatal bladder management	4.72 ± 0.57	0.12
1.1 At admission, obtain the pregnant woman’s history of urinary system and general information, including: presence of urinary tract infection symptoms within the past week, fluid intake, time since last normal void, history of voiding and defecation abnormalities, and surgical history.	4.67 ± 0.59	0.13
1.2 At admission, perform abdominal examination to assess the degree of bladder distension and the sensation of bladder filling.	4.50 ± 0.79	0.17
1.3 Instruct pelvic floor muscle training, recommending that it begin at 28 weeks of gestation and continue for at least 6 weeks, with a total daily training time of 15 min. Pelvic floor muscle training should initially be performed lying down or sitting, then gradually transition to standing and during activity. Two types of exercises are recommended to train both fast-twitch and slow-twitch muscles: the first is sustained contractions (hold as long as possible, generally 6–8 s), and the second is quick contractions with rapid release. During exercise, avoid breath-holding, limit compensatory use of gluteal and abdominal muscles, and ensure adequate relaxation between contractions. It is recommended to combine diaphragmatic breathing with pelvic floor muscle training: relax the pelvic floor during deep inhalation and contract it during deep exhalation.	4.83 ± 0.38	0.08
1.4 Encourage pregnant women (except those with strict fluid restrictions) to drink 1.5–2 L of fluid daily during pregnancy. Limit caffeine and carbonated beverages, and avoid alcohol.	4.72 ± 0.57	0.12
1.5 During pregnancy, educate women on how to prevent urinary tract infections and inform them about changes in bladder function during pregnancy and childbirth.	4.83 ± 0.38	0.08
2. Intrapartum bladder management	5.00 ± 0.00	0.00
2.1 For women with high-risk factors (e.g., history of voiding abnormalities, relevant surgical history, etc.), complete a fluid balance chart recording all oral and intravenous fluids and voided volumes, and note the use of oxytocin.	4.61 ± 0.50	0.11
2.2 Encourage voiding every 2 h and observe the colour, characteristics, and volume of urine.	4.89 ± 0.32	0.07
2.3 If the woman cannot void every 2 h, assess fluid intake and bladder capacity, and consider intermittent or indwelling catheterisation as needed.	4.94 ± 0.24	0.05
2.4 For women who cannot void spontaneously, the frequency of intermittent catheterisation during labour should be at least every 4 h; if a single catheterised volume exceeds 500 mL, the interval should be shortened.	4.78 ± 0.43	0.09
2.5 Before entering the second stage of labour, if the woman is judged suitable for vaginal delivery and has an indwelling catheter, the catheter should be removed to avoid urethral injury.	4.83 ± 0.38	0.08
2.6 If the woman has received neuraxial labour analgesia, healthcare providers should assess whether her sensation of bladder filling is diminished or lost.	5.00 ± 0.00	0.00
3. Postpartum bladder management	5.00 ± 0.00	0.00
3.1 Advise women to start pelvic floor muscle training (e.g., Kegel exercises) as soon as possible after delivery according to their individual condition, and to progress gradually to strengthen pelvic floor function.	4.83 ± 0.38	0.08
3.2 After delivery, encourage the woman to void as soon as possible. If she has not voided before leaving the delivery room, inform the postpartum ward staff so that the time and volume of the first void can be accurately recorded.	5.00 ± 0.00	0.00
3.3 After catheter removal, healthcare providers should assess and document the woman’s voiding attempts, sensation of voiding, and bladder function.	4.94 ± 0.24	0.05
3.4 Record the first two voids after delivery or after catheter removal for all women. Women should measure their voided volumes and report them to staff.	5.00 ± 0.00	0.00
3.5 Ensure that adequate pain relief and mobility are in place before the first void/catheter removal.	4.72 ± 0.57	0.12
3.6 Actively encourage all women to void every 2 h after delivery or after catheter removal.	4.78 ± 0.43	0.09
3.7 Ensure adequate pain relief, inspect the perineum for any swelling or other abnormalities, and recommend cold compresses.	4.89 ± 0.32	0.07
3.8 Manage constipation as needed.	4.56 ± 0.51	0.11
3.9 Ensure adequate fluid intake (1.5–2 L per day) for women; encourage moderate fluid intake at 2 h postpartum to avoid rapid overdistension of the bladder.	4.89 ± 0.32	0.07
3.10 Ensure that the first void occurs within 4 to 6 h after delivery. If the woman has not voided within 4 h postpartum or after catheter removal, initiate 2 h of conservative measures (e.g., adequate analgesia, fluid replacement, privacy, warm sitz baths, running water sounds, hot compresses and massage over the bladder area, perineal irrigation, acupuncture at relevant points, etc.) and start strict fluid balance charting. If signs of acute urinary retention appear within 4 h after delivery or catheter removal, intervention should not be delayed for an additional 2 h. Intramuscular neostigmine may be considered when conservative measures fail and no contraindications exist. Contraindicated in bronchial asthma; use with caution in hyperthyroidism and Parkinson’s disease. Adverse effects include nausea, vomiting, diarrhea, and bradycardia. This is an off-label clinical extension of its approved indication for postoperative urinary retention. pharmacological options should be clearly distinguished from non-pharmacological measures and used only when benefits outweigh risks, with individualized clinical assessment. If signs of acute urinary retention appear within 4 h after delivery or catheter removal, intervention should not be delayed for an additional 2 h.	4.94 ± 0.24	0.05
3.11 If conservative measures fail within 6 h after delivery, assess the woman for urinary retention and dehydration: measure post-void residual volume using catheterisation or real-time bladder scanner within 10 min after voiding; residual volume > 150 mL is considered urinary retention. This threshold is a clinical reference rather than an absolute diagnostic criterion and that management decisions should consider symptoms, bladder function, and clinical assessment.	4.83 ± 0.51	0.11
3.12 If bladder overdistension > 600 mL occurs, report to the senior clinician and manage actively.	4.94 ± 0.24	0.05
3.13 If the woman has normal fluid intake, two spontaneous voids of ≥200 mL each, no bladder distension on examination, and normal bladder sensation, no further action is required.	4.78 ± 0.43	0.09
4. Management of urinary retention	5.00 ± 0.00	0.00
4.1 If available, a bladder scanner should be preferred over indwelling catheterisation as the first choice for measuring post-void residual volume.	4.89 ± 0.32	0.07
4.2 If 150 mL ≤ residual volume < 500 mL, leave an indwelling urinary catheter for continuous drainage for 24 h. If residual volume is ≥500 mL but <1000 mL, leave the catheter for continuous drainage for 48 h. If residual volume is ≥1000 mL, leave the catheter for continuous drainage for 3–5 days. There is no consensus on the optimal timing for catheter removal. Clinical judgment is particularly important in this regard. Reassess bladder function at least daily and consider early catheter removal when clinically appropriate.	4.61 ± 0.78	0.17
4.3 Inform the senior obstetrician, nurse in charge, and midwife of the acute episode of urinary retention and involve them in ongoing care.	4.83 ± 0.38	0.08
4.4 Catheters should be removed in the morning, preferably after 6 a.m. If removal is expected after 6 p.m., it is recommended to leave the catheter in place until the next morning to facilitate measurement of the subsequent two voids.	4.67 ± 0.59	0.13
4.5 If the woman is to be discharged with a catheter in place, clearly specify the date for return for a trial without catheter (TWOC) and ensure she has received adequate education on bladder management and catheter care, including fluid intake, avoidance of constipation, perineal hygiene, hand hygiene, and catheter care.	4.94 ± 0.24	0.05

## Data Availability

The data that support the findings of this study are available from the corresponding author. The data are not publicly available due to privacy restrictions.

## Supplementary Materials

The following supporting information can be downloaded at: https://www.mdpi.com/article/10.3390/healthcare14213773/s1, Table S1: Literature search strategy and results.
