# Supplementary material for: Development of a Complex Intervention for Perinatal Bladder Management Among Pregnant Women Based on the Medical Research Council Framework: A Development Study
Source: Healthcare (Basel). 2026 Nov 4;14(21):3773. doi: 10.3390/healthcare14213773 (PMC13762874; doi:10.3390/healthcare14213773)
Supplement: Supplementary file 1 [file healthcare-14-03773-s001.zip › healthcare-4584939-supplementary.pdf]

Supplementary Materials

Table S1: Literature search strategy and results

PubMed(943):

#1	"Pregnant People"[MeSH Terms] OR "Pregnant Women"[MeSH Terms]	16350
#2	"People, Pregnant"[Title/Abstract] OR "Pregnant Peoples"[Title/Abstract] OR "Pregnant Person"[Title/Abstract] OR "Pregnant Woman"[Title/Abstract] OR "Woman, Pregnant"[Title/Abstract] OR "Women, Pregnant"[Title/Abstract] OR "Pregnant Women"[Title/Abstract] OR "gravida"[Title/Abstract] OR "gestation"[Title/Abstract] OR "pregnancy"[Title/Abstract] OR "puerpera"[Title/Abstract] OR "postpartum"[Title/Abstract] OR "intrapartum"[Title/Abstract] OR "postnatal"[Title/Abstract]	794842
#3	"Urinary Bladder"[MeSH Terms] OR "Urinary Catheters"[MeSH Terms] OR "Pelvic Floor"[MeSH Terms] OR "Massage"[MeSH Terms]	71627
#4	"Bladder, Urinary"[Title/Abstract] OR "Bladder"[Title/Abstract] OR "Bladder Detrusor Muscle"[Title/Abstract] OR "Bladder Detrusor Muscles"[Title/Abstract] OR "Detrusor Muscle, Bladder"[Title/Abstract] OR "Detrusor Muscles, Bladder"[Title/Abstract] OR "Detrusor Urinae"[Title/Abstract] OR "Catheters, Urinary"[Title/Abstract] OR "Catheter, Urinary"[Title/Abstract] OR "Urinary Catheter"[Title/Abstract] OR "Ureteral Catheters"[Title/Abstract] OR "Catheters, Ureteral"[Title/Abstract] OR "Catheter, Ureteral"[Title/Abstract] OR "Ureteral Catheter"[Title/Abstract] OR "Urethral Catheters"[Title/Abstract] OR "Catheters, Urethral"[Title/Abstract] OR "Catheter, Urethral"[Title/Abstract] OR "Urethral Catheter"[Title/Abstract] OR "Floor, Pelvic"[Title/Abstract] OR "Pelvic Diaphragm"[Title/Abstract] OR "Diaphragm, Pelvic"[Title/Abstract] OR "Diaphragms, Pelvic"[Title/Abstract] OR "Pelvic Diaphragms"[Title/Abstract] OR "Zone Therapy"[Title/Abstract] OR "Therapies, Zone"[Title/Abstract] OR "Zone Therapies"[Title/Abstract] OR "Therapy, Zone"[Title/Abstract] OR "Massage Therapy"[Title/Abstract] OR "Massage Therapies"[Title/Abstract] OR "Therapies, Massage"[Title/Abstract] OR "Therapy, Massage"[Title/Abstract] OR "Bladder management"[Title/Abstract] OR "Perineum"[Title/Abstract] OR "perineum irrigation"[Title/Abstract] OR "Pelvic floor muscles"[Title/Abstract] OR "Pelvic floor exercises"[Title/Abstract] OR "void"[Title/Abstract] OR "ice pack"[Title/Abstract] OR "cold pack"[Title/Abstract] OR "hot pack"[Title/Abstract] OR "foment"[Title/Abstract] OR "hot compress*"[Title/Abstract] OR "cold compress*"[Title/Abstract]	223735
#5	"Urinary Retention"[MeSH Terms]	5418
#6	"Retention, Urinary"[Title/Abstract]	113

#7	"Medicine, Chinese Traditional"[MeSH Terms] OR "Medicine"[MeSH Terms] OR "Acupuncture Therapy"[MeSH Terms]	1359506
#8	"Zhong Yi Xue"[Title/Abstract] OR "Chung I Hsueh"[Title/Abstract] OR "Hsueh, Chung I"[Title/Abstract] OR "Traditional Medicine, Chinese"[Title/Abstract] OR "Chinese Traditional Medicine"[Title/Abstract] OR "Traditional Chinese Medicine"[Title/Abstract] OR "Chinese Medicine, Traditional"[Title/Abstract] OR "Traditional Tongue Diagnosis"[Title/Abstract] OR "Tongue Diagnoses, Traditional"[Title/Abstract] OR "Tongue Diagnosis, Traditional"[Title/Abstract] OR "Traditional Tongue Diagnoses"[Title/Abstract] OR "Traditional Tongue Assessment"[Title/Abstract] OR "Tongue Assessment, Traditional"[Title/Abstract] OR "Traditional Tongue Assessments"[Title/Abstract] OR "Specialties, Medical"[Title/Abstract] OR "Medical Specialties"[Title/Abstract] OR "Medical Specialty"[Title/Abstract] OR "Specialty, Medical"[Title/Abstract] OR "Specialities, Medical"[Title/Abstract] OR "Medical Speciality"[Title/Abstract] OR "Speciality, Medical"[Title/Abstract] OR "Medical Specialities"[Title/Abstract] OR "Insurance Medicine"[Title/Abstract] OR "Insurance Medicines"[Title/Abstract] OR "Medicine, Insurance"[Title/Abstract] OR "Medicines, Insurance"[Title/Abstract] OR "Acupuncture Treatment"[Title/Abstract] OR "Acupuncture Treatments"[Title/Abstract] OR "Treatment, Acupuncture"[Title/Abstract] OR "Therapy, Acupuncture"[Title/Abstract] OR "Pharmacoacupuncture Treatment"[Title/Abstract] OR "Treatment, Pharmacoacupuncture"[Title/Abstract] OR "Pharmacoacupuncture Therapy"[Title/Abstract] OR "Therapy, Pharmacoacupuncture"[Title/Abstract] OR "Acupotomy"[Title/Abstract] OR "Acupotomies"[Title/Abstract]	55048
#9	"Guideline"[Publication Type] OR "Guidelines as Topic"[MeSH Terms] OR "Systematic Review"[Publication Type] OR "Systematic Reviews as Topic"[MeSH Terms] OR "randomized controlled trial"[Publication Type] OR "controlled clinical trial"[Publication Type] OR "clinical trial"[Publication Type] OR "Clinical Trials as Topic"[MeSH Terms] OR "Randomized Controlled Trials as Topic"[MeSH Terms] OR "Controlled Clinical Trials as Topic"[MeSH Terms] OR "Consensus Development Conferences as Topic"[Mesh] OR "Consensus Development Conference"[Publication Type] OR "Meta-Analysis as Topic"[MeSH Terms] OR "Meta-Analysis"[Publication Type] OR "Practice Guideline"[Publication Type] OR "Practice Guidelines as Topic"[MeSH Terms]	1863670
#10	"guideline"[Title/Abstract] OR "meta-analysis"[Title/Abstract] OR "Expert consensus"[Title/Abstract] OR "Meta-synthesis"[Title/Abstract]	891365

	OR "Evidence Summary"[Title/Abstract] OR "Systematic Review"[Title/Abstract] OR "randomized controlled trial"[Title/Abstract] OR "clinical trial"[Title/Abstract] OR "Consensus Development Conferences"[Title/Abstract] OR "Controlled Clinical Trials"[Title/Abstract]	
#11	(((#1 OR #2) AND (#3 OR #4 OR #5 OR #6)) OR ((#5 OR #6) AND (#7 OR #8))) AND (#9 OR #10)	943

Embase(1458):

#1	'pregnant woman'/exp	129178
#2	gravida:ti,ab,kw OR gestation:ti,ab,kw OR pregnancy:ti,ab,kw OR puerpera:ti,ab,kw OR postpartum:ti,ab,kw OR intrapartum:ti,ab,kw OR postnatal:ti,ab,kw	1000128
#3	'bladder and bowel management'/exp OR 'urinary catheter'/exp OR 'pelvic floor muscle training'/exp OR 'perineal massage'/exp OR 'indwelling catheter'/exp OR 'intermittent catheterization'/exp OR 'ice pack'/exp OR 'cold compress'/exp	51747
#4	'perineum irrigation':ti,ab,kw OR void:ti,ab,kw OR 'hot compress':ti,ab,kw OR foment:ti,ab,kw	25363
#5	'urine retention'/exp	
#6	'Chinese medicine'/exp OR 'medicine'/exp OR 'moxibustion'/exp OR 'acupuncture point'/exp	4493624
#7	'zhongyixue':ti,ab,kw OR 'chung i hsueh':ti,ab,kw	3
#8	'clinical practice guideline'/exp OR 'practice guideline'/exp OR 'systematic review'/exp OR 'systematic review (topic)'/exp OR 'meta analysis'/exp OR 'meta analysis (topic)'/exp OR 'randomized controlled trial'/exp OR 'randomized controlled trial (topic)'/exp OR 'controlled clinical trial'/exp OR 'controlled clinical trial (topic)'/exp OR 'consensus development'/exp	2573360
#9	'evidence summary':ti,ab,kw OR guideline:ti,ab,kw OR 'meta analysis':ti,ab,kw OR 'expert consensus':ti,ab,kw OR 'meta synthesis':ti,ab,kw OR 'systematic review':ti,ab,kw OR 'randomized controlled trial':ti,ab,kw OR 'clinical trial':ti,ab,kw OR 'consensus development conferences':ti,ab,kw OR 'controlled clinical trials':ti,ab,kw	1221872
#10	(((#1 OR #2) AND (#3 OR #4 OR #5)) OR (#5 AND (#6 OR #7))) AND (#8 OR #9)	1458

Web of Science(1371):

#1	Pregnant People OR Pregnant Person OR Pregnant Woman OR Women, Pregnant OR Pregnant Women OR postpartum OR intrapartum OR postnatal	617679
#2	Urinary Catheters OR Ureteral Catheters OR Urethral Catheters OR Pelvic Floor OR Massage OR Massage Therapy OR Therapy, Zone OR Zone	563589

	Therapy OR Catheters, Indwelling OR Implantable Catheters OR In-Dwelling Catheters OR Pelvic floor exercises OR Pelvic floor muscles OR Bladder management OR perineum irrigation OR ice pack OR cold pack OR hot pack OR foment OR hot compress OR cold compress OR intermittent catheterization	
#3	Urinary Retention	41921
#4	Medicine, Chinese Traditional OR Chinese Medicine, Traditional OR Chinese Traditional Medicine OR Chung I Hsueh OR Traditional Chinese Medicine OR Traditional Medicine, Chinese OR Traditional Tongue Assessment OR Traditional Tongue Diagnosis OR Zhong Yi Xue OR Medicine OR Insurance Medicine OR Medical Specialities OR Medical Specialties OR Medical Specialty OR Specialities, Medical OR Specialties, Medical OR Specialty, Medical OR Moxibustion OR Moxabustion OR Acupuncture	11790553
#5	Practice Guideline OR Clinical Guideline OR Clinical Guidelines OR Clinical Practice Guideline OR Intervention Guideline OR Therapeutic Guideline OR Treatment Guideline OR Guideline OR Systematic Review OR Review, Systematic OR Umbrella Review OR Guidelines as Topic OR Systematic Reviews as Topic OR Reviews Systematic as Topic OR Systematic Review as Topic OR Umbrella Reviews as Topic OR Randomized Controlled Trial OR Randomized Controlled Trials as Topic OR Clinical Trials, Randomized OR Controlled Clinical Trials, Randomized OR Trials, Randomized Clinical OR Consensus Development Conferences as Topic OR Conferences, Consensus Development as Topic OR Conferences, Consensus Statement as Topic OR Consensus Statement Conferences as Topic OR Consensus Workshop OR Consensus Workshops OR Workshop, Consensus OR Workshops, Consensus OR Consensus Development Conference OR Consensus Statement Conference OR Meta-Analysis as Topic OR Clinical Trial Overviews OR Overviews, Clinical Trial OR Meta-Analysis OR Meta-synthesis OR Expert consensus OR Evidence Summary	3147357
#6	TS=((((#1) AND (#2 OR #3)) OR ((#1) AND (#3) AND (#4))) AND (#5))	1371

Cochrane library(624):

#1	MeSH descriptor: [Pregnant People] explode all trees	990
#2	(Pregnant Peoples OR People, Pregnant OR Pregnant Person OR Pregnant Woman OR Pregnant Women OR Woman, Pregnant OR Women, Pregnant OR postpartum OR intrapartum OR postnatal):ti,ab,kw	41683
#3	MeSH descriptor: [Urinary Catheters] explode all trees	175
#4	MeSH descriptor: [Pelvic Floor] explode all trees	990
#5	MeSH descriptor: [Massage] explode all trees	1689
#6	MeSH descriptor: [Catheters, Indwelling] explode all trees	1256
#7	(Urinary Catheter OR Catheter, Urinary OR Catheters, Urinary OR Urethral	18042

	Catheter OR Urethral Catheters OR Catheter, Urethral OR Catheters, Urethral OR Catheter, Ureteral OR Ureteral Catheter OR Ureteral Catheters OR Catheters, Ureteral OR Therapies, Zone OR Therapy, Zone OR Zone Therapies OR Zone Therapy OR Massage Therapies OR Therapies, Massage OR Massage Therapy OR Therapy, Massage OR Implantable Catheters OR In Dwelling Catheters OR In-Dwelling Catheters OR Indwelling Catheters OR In-Dwelling Catheter OR Catheter, In-Dwelling OR Catheter, Indwelling OR Indwelling Catheter OR Catheters, In-Dwelling OR Pelvic floor exercises OR Pelvic floor muscles OR Bladder management OR perineum irrigation OR ice pack OR cold pack OR hot pack OR foment OR hot compress OR cold compress OR intermittent catheterization):ti,ab,kw	
#8	MeSH descriptor: [Urinary Retention] explode all trees	607
#9	(Retention, Urinary):ti,ab,kw	4437
#10	MeSH descriptor: [Medicine, Chinese Traditional] explode all trees	1791
#11	MeSH descriptor: [Medicine] explode all trees	27419
#12	MeSH descriptor: [Moxibustion] explode all trees	681
#13	MeSH descriptor: [Acupuncture Points] explode all trees	2794
#14	(Traditional Medicine, Chinese OR Chinese Traditional Medicine OR Traditional Chinese Medicine OR Chung I Hsueh OR Chinese Medicine, Traditional OR Zhong Yi Xue OR Hsueh, Chung I OR Tongue Diagnoses, Traditional OR Tongue Diagnosis, Traditional OR Traditional Tongue Assessments OR Tongue Assessment, Traditional OR Traditional Tongue Assessment OR Traditional Tongue Diagnosis OR Traditional Tongue Diagnoses OR Insurance Medicine OR Insurance Medicines OR Medicines, Insurance OR Medicine, Insurance OR Medical Specialties OR Specialty, Medical OR Medical Specialty OR Medical Specialities OR Medical Speciality OR Specialties, Medical OR Specialities, Medical OR Speciality, Medical OR Moxabustion OR Acupoint OR Point, Acupuncture OR Acupuncture Point OR Points, Acupuncture OR Acupoints):ti,ab,kw	24884
#15	MeSH descriptor: [Systematic Review] explode all trees	1
#16	MeSH descriptor: [Systematic Reviews as Topic] explode all trees	134
#17	MeSH descriptor: [Randomized Controlled Trial] explode all trees	34
#18	MeSH descriptor: [Randomized Controlled Trials as Topic] explode all trees	59274
#19	MeSH descriptor: [Consensus Development Conferences as Topic] explode all trees	42
#20	MeSH descriptor: [Meta-Analysis as Topic] explode all trees	1487
#21	MeSH descriptor: [Meta-Analysis] explode all trees	92
#22	(Guideline OR Expert consensus OR Meta-synthesis OR Evidence Summary OR Systematic Review OR randomized controlled trial OR meta-analysis OR Consensus Development Conference):ti,ab,kw	799726
#23	(((#1 OR #2) AND (#3 OR #4 OR #5 OR #6 OR #7 OR #8 OR #9)) OR ((#8 OR #9) AND (#10 OR #11 OR #12 OR #13 OR #14))) AND (#15 OR #16 OR #17 OR #18 OR #19 OR #20 OR #21 OR #22)	624

CINAHL(456):

#1	SU pregnancy	234201
#2	XB Pregnant People OR Pregnant Person OR Pregnant Woman OR Women, Pregnant OR Pregnant Women OR postpartum OR intrapartum OR postnatal	96539
#3	SU Bowel and Bladder Management OR Catheters Urinary OR pelvic floor muscles OR kegel exercises OR massage OR urinary catheterization,intermittent	29798
#4	XB indwelling catheter OR Pelvic floor exercises OR Pelvic floor muscles OR Bladder management OR perineum irrigation OR ice pack OR cold pack OR hot pack OR foment OR hot compress OR cold compress	6664
#5	SU urinary retention	1819
#6	XB Bladder, Distended OR Distended Bladder OR Urinary Retaining	74
#7	SU medicine,Chinese traditional OR medicine OR Moxibustion OR Acupuncture	12980
#8	XB Chinese Medicine OR Chinese Medicine, Traditional OR Chinese Traditional Medicine OR Medicine, Chinese OR Medicine, Traditional Chinese OR Traditional Chinese Medicine OR Traditional Medicine, Chinese OR Medicines OR Moxabustion OR Acupuncture Therapy	204340
#9	SU Systematic Review OR practice guidelines OR Meta-Analysis OR Randomized Controlled Trials OR Consensus	404989
#10	XB Integrative Review OR Integrative Reviews OR Systematic Reviews OR Clinical Practice Guidelines OR Practice Guideline OR Meta Analyses OR Metaanalyses OR Metaanalysis OR Clinical Trial, Randomized OR Clinical Trials, Controlled OR Randomized, Clinical Trials OR Randomized, Controlled Clinical Trials OR Randomized, Controlled Trials OR Randomized, Randomised Controlled Trial OR Randomised Controlled Trials OR Randomized Clinical Trial OR Randomized Clinical Trials OR Randomized Controlled Clinical Trials OR Randomized Controlled Trial OR Consensus Development OR Group Consensus OR Expert consensus OR Meta-synthesis OR Evidence Summary OR Systematic Review OR practice guidelines OR Meta-Analysis OR Randomized Controlled Trials OR Consensus	449210
#11	(((#1 OR #2) AND (#3 OR #4 OR #5 OR #6)) OR ((#5 OR #6) AND (#7 OR #8))) AND (#9 OR #10)	456

CNKI 89：

(((SU=孕妇+ 孕产妇健康+ 围产期 OR TKA=产前+ 产时+ 产后 + 孕产妇+ 产妇) AND (SU=间歇导尿+ 热敷疗法+ 按摩 OR TKA=会阴冲洗+ 诱导排尿+ 盆底肌锻炼+ 凯格尔运动+ 留置导尿+ 膀胱管理 OR SU=尿潴留)) OR (SU=尿潴留 AND TKA=中医+ 针灸+ 穴位 + 药物)) AND (SU=系统评价（主题）+ 随机对照试验（主题）+ 指南+ meta 分析（主题） OR TKA=系统评价 +证据总结+ Meta 分析+ 随机对照试验+ 指南+ 专家共识+ meta 整合)

Wanfang database　89：

(((主题:(孕妇 OR 孕产妇健康 OR 围产期)OR 题名或关键词:(产前 OR 产时 OR 产后 OR 孕产妇 OR 产妇)) AND (主题:(间歇导尿 OR 热敷疗法 OR 按摩)OR 题名或关键词:(会阴冲洗 OR 诱导排尿 OR 盆底肌锻炼 OR 凯格尔运动 OR 留置导尿 OR 膀胱管理)OR 主题:(尿潴留))) OR (主题:(尿潴留)AND 题名或关键词:(中医 OR 针灸 OR 穴位 OR 药物)))AND (主题:(系统评价（主题）OR 随机对照试验（主题）OR 指南 OR meta 分析（主题）)OR 题名或关键词:(系统评价 OR 证据总结 OR Meta 分析 OR 随机对照试验 OR 指南 OR 专家共识 OR meta 整合))

SinoMed　615：

((((孕妇[常用字段:智能] OR 孕产妇健康[常用字段:智能] OR 围产期[常用字段:智能])OR (产前[常用字段:智能] OR 产时[常用字段:智能] OR 产后[常用字段:智能]OR 孕产妇[常用字段:智能] OR 产妇[常用字段:智能])) AND ((间歇导尿[常用字段:智能] OR 热敷疗法[常用字段:智能] OR 按摩[常用字段:智能])OR (会阴冲洗[常用字段:智能] OR 诱导排尿[常用字段:智能] OR 盆底肌锻炼[常用字段:智能] OR 凯格尔运动[常用字段:智能] OR 留置导尿[常用字段:智能] OR 膀胱管理[常用字段:智能])OR (尿潴留[常用字段:智能]))) OR ((尿潴留[常用字段:智能]) AND (中医[常用字段:智能]OR 针灸[常用字段:智能] OR 穴位[常用字段:智能] OR 药物[常用字段:智能]))) AND ((系统评价（主题）[常用字段:智能] OR 随机对照试验（主题）[常用字段:智能] OR 指南[常用字段:智能] OR meta 分析（主题）[常用字段:智能]) OR (系统评价[常用字段:智能] OR 证据总结[常用字段:智能] OR Meta 分析[常用字段:智能] OR 随机对照试验[常用字段:智能] OR 指南[常用字段:智能] OR 专家共识[常用字段:智能] ORmeta 整合[常用字段:智能]))
